# Unpaid Caregiving and Labor Force Participation among Chinese Middle-Aged Adults

**DOI:** 10.3390/ijerph18020641

**Published:** 2021-01-13

**Authors:** Huamin Chai, Rui Fu, Peter C. Coyte

**Affiliations:** 1 School of Public Administration, East China Normal University, Shanghai 200241, China; huaminchai@126.com; 2 Center for Public Policy Research, East China Normal University, Shanghai 200241, China; 3 Institute of Health Policy, Management and Evaluation, Dalla Lana School of Public Health, University of Toronto, Toronto, ON M5T 3M6, Canada; peter.coyte@utoronto.ca

**Keywords:** family caregivers, labor force, labor supply, employment, China

## Abstract

Unpaid family caregivers must consider the economic trade-off between caregiving and paid employment. Prior literature has suggested that labor force participation (LFP) declines with caregiving intensity, but no study has evaluated this relationship by accounting for the presence of both kinks and discontinuities. Here we used respondents of the China Health and Retirement Longitudinal Study baseline survey who were nonfarming, of working age (aged 45–60) and had a young grandchild and/or a parent/parent-in-law. For women and men separately, a caregiving threshold-adjusted probit model was used to assess the association between LFP and weekly unpaid caregiving hours. Instrumental variables were used to rule out the endogeneity of caregiving hours. Of the 3718 respondents in the analysis, LFP for men was significantly and inversely associated with caregiving that involved neither discontinuities nor kinks. For women, a kink was identified at the caregiving threshold of eight hrs/w such that before eight hours, each caregiving hour was associated with an increase of 0.0257 in the marginal probability of LFP, but each hour thereafter was associated with a reduction of 0.0014 in the marginal probability of LFP. These results have implications for interventions that simultaneously advance policies of health, social care and labor force.

## 1. Introduction

### 1.1. Background

With a rapidly ageing population and an increasing presence of women in the labor force, societies face a crisis when confronted with a dramatic increase in the demand for caregiving. In many low- or middle-income countries, middle-aged adults are often unpaid caregivers for their ageing parents (or parents-in-law), whereas grandparents usually take on the parental role for their grandchildren [1,2]. 

An important case study of unpaid caregiving among family members is China, which, due to decades of falling birth rates and rising life expectancy, has the world’s fastest ageing population [3]. Recent data suggest that 90% of Chinese families are in need of either elderly care or childcare while 40% of them require both [4]. Because China has very limited publicly funded home care, the percentage of adults who are unpaid caregivers to one or more family members is very high. Specifically, studies show that 57%, 40%, 38% and 27% of daughters, sons, daughters-in-law and sons-in-law regularly provide care to elderly parents (or in-laws) [5] and 70% of grandparents raise their grandchildren [6]. It is very important to note that grandparenthood generally occurs early in the life course of Chinese adults because an average of 80% of them become grandparents by the age of 55 [7]. This means middle-aged Chinese adults (between 45–60 years) are very likely to assume multiple caregiving roles and must consider the relevant economic trade-off between providing unpaid familiar care and engaging in paid employment. 

### 1.2. Literature Review

The relationship between the intensity of unpaid caregiving and the probability of labor force participation (LFP) has been studied extensively in the international literature. A consensus has emerged from these studies that suggests LFP is generally inversely related to more intensive caregiving and that there exists a caregiving threshold beyond which increased caregiving has a larger negative effect on LFP than before that threshold is reached [8]. What remains unclear from the literature is the exact form of such caregiving thresholds, specifically if they constitute kinks in and/or discontinuities to the relationship between caregiving and LFP. Most studies have only located discontinuities but did not conduct analyses to identify potential kinks. Carmichael and Charles [9,10] found two simultaneous discontinuities at caregiving hours CG > 0 and at CG > 10 hrs/w; Heitmueller [11] assessed a single discontinuity at CG > 20 hrs/w following a prior study [12] that had suggested that threshold; Van Houtven et al [2] tested a single discontinuity at either CG > 0 or CG > 20 hrs/w, and Jacobs and colleagues [13,14] examined multiple discontinuities at 0, 5 and 15 hrs/w. Meanwhile, several studies have estimated kinks by testing a set of rather arbitrary thresholds (10, 15 and 20 hrs/w), but they did not assess potential discontinuities at these thresholds [15,16]. To date, no study has simultaneously examined both kinks and discontinuities associated with the relationship between LFP and unpaid caregiving; indeed, neither term (“kink” nor “discontinuity”) was specifically mentioned in the referenced articles [8].

This paper aims to fill this gap in the empirical literature. By using nationally representative data from the China Health and Retirement Longitudinal Study baseline survey, we investigated potential thresholds of weekly unpaid caregiving hours to see if they resulted in kinks and/or discontinuities in the relationship between caregiving and labor force participation. Our results will provide actionable implications for policy strategies that support family caregivers to balance their caregiving activities and work commitment.

### 1.3. Conceptual Framework

In the standard labor-leisure choice theory, individuals with family responsibilities must simultaneously allocate time to labor work, leisure and unpaid home care to maximize their utility [17,18,19]. Depending on the number of hours of caregiving, individuals confront the traditional labor-leisure trade-off wherein the decision of whether or not to work depends on the reservation wage, nonwage income and an array of sociodemographic and other contextual variables. Theory predicts that when holding all else constant, an increase in caregiving hours reduces the maximum amount of time available for paid work and thereby induces an availability effect that tends to lower LFP. The resulting relationship between caregiving intensity and LFP depends on a host of intrinsic factors including preference orderings between labor and leisure, social context and the life cycle [20]. Gendered impacts on the choices between caregiving and LFP are predicted to be in line with economic theories of specialization and bargaining [18,21] which may be particularly salient in the case of caring for certain dependents (such as parents or spouse) [22].

Recent work by Van Houtven and colleagues [8] advances the theory by examining the theoretical basis for a caregiving hours threshold. This work assesses labor-leisure preference orderings in which the marginal rate of substitution may change abruptly if leisure time were below a leisure threshold. As people value an incremental leisure time before this threshold more than they do after the threshold, this allows for the possibility that kinks may arise on indifference curves thereby yielding possible kinks and/or discontinuities in the relationship between caregiving hours and LFP.

## 2. Materials and Methods 

Our empirical work was informed by this theoretical model in order to test for a potentially nonlinear relationship between hours of caregiving and LFP by accounting for possible presence of both kinks and discontinuities. Analysis was conducted on Chinese men and women separately to reflect the gender gap in employment [23]. In the following sections, we describe the data sources (Section 2.1.); the study sample (Section 2.2.); present the variables used in the analysis (Section 2.3.); and detail the statistical procedures, including identifying a caregiving threshold, testing for endogeneity of caregiving hours using instrumental variables (Section 2.4.) and sensitivity analyses (Section 2.5.).

### 2.1. Study Setting and Design

This is a population-based cross-sectional study using person-level data from the China Health and Retirement Longitudinal Study (CHARLS) baseline survey. CHARLS is a nationally representative panel study designed to comprehensively understand social trends, socio-economic well-being and the ageing of community-dwelling Chinese adults aged 45 or older. Between June 2011 and March 2012, a nationally representative sample of 23,422 dwelling units representing potential households was drawn from 150 counties (or districts) and 450 villages (or communities) in 28 provinces across China using multi-stage probability sampling [24]. Excluding empty or non-resident dwelling units yielded 12,740 age-eligible households for the baseline survey. Within each sampled household, the main respondent, defined to be a family member who was at least 45 years of age with sufficient knowledge about the household, and his/her spouse (if any) were both invited to participate in the baseline survey. This procedure yielded 10,257 households with at least one age-eligible member who agreed to participate in the baseline survey (response rate = 80.5%). A total of 17,708 individuals from these households completed the baseline survey at home using a face-to-face computer-assisted personal interview technique [24].

### 2.2. Study Sample

The analysis included CHARLS baseline participants who were of working age; not engaged in agricultural work or in an unpaid family business; and had at least one grandchild under the age of 16 or a parent (or parent-in-law) that was still alive. The normal pensionable age in China is 60 for males, 55 for white-collar women (such as teachers and civil servants) and 50 for blue-collar women [25]. Consequently, in order to consider the potential trade-off between paid work and caregiving, we limited the sample to only men aged between 45–60 and women aged between 45–55 (*N* = 8,603, 48.6%). We excluded participants who reported being either agricultural workers, unpaid family business workers (*N* = 4,140); self-employed individuals who worked with another hired family employee (*N* = 264); those who did not report having grandchildren under the age of 16 or parents (or parents-in-law) that were alive (*N* = 368); or those who had missing data in the survey (*N* = 113). These procedures resulted in 3718 (21.0% of the total sample) participants who met the eligibility criteria, which comprised 2,268 men (61.0%) and 1,450 women (39.0%). 

### 2.3. Variables

We used a binary outcome variable to represent individual’s self-reported current labor force participation (LFP) status using the survey question, “Did you work for at least one-hour last week? We consider any of the following activities to be work: earn a wage, run your own business and unpaid family business work, et al. Work does not include doing your own housework or doing activities without pay, such as voluntary work.” Those who answered “Yes”, were classified as labor force participants while those who responded “No”, were non-participants [26].

The primary exposure variable was the number of hrs/w an individual provided unpaid caregiving services to grandchildren, parents and/or parents-in-law without financial compensation in the last year. In the survey, individuals reported how many hrs/w in the past year they had cared for each dependent (grandchildren, parents and parents-in-law). These responses were summed to yield total weekly unpaid caregiving hours. Those who did not report any caregiving activity over the past year were assigned a value of 0. 

We extracted the following person-level characteristics from the survey that previously were identified in the literature [2,27,28,29] to influence the decisions to participate in the labor market and the intensity of caregiving at home: age (years), marital status (currently married vs. not married), highest education (illiterate or primary/elementary school, middle school, high school or college and above), area of residence (urban vs. rural), having work-limiting health conditions (yes vs. no), household size (i.e., numbers of people in the household; between 1–12) and monthly income of spouse. In sensitivity analysis, we introduced four more covariates, including whether the individual held an urban or rural Hukou (household registration), the household income, the place of Hukou registration in terms of the three economic macro-regions (in the East, Central or West China) [30,31,32] and the tier of city where respondents had registered their Hukou on the basis of the 2013 version of China’s City-Tier Classification (in a city that was ranked Tier 2 or above vs. a city below Tier 2) [33].

### 2.4. Statistical Analysis

We first compared the baseline characteristics of respondents stratified by their labor force participation (LFP) status using two-sample tests (t-test or Chi-square test). Probit regression analysis was performed for women and men separately to estimate the association between weekly caregiving hours and LFP. In each analysis, we investigated a potential threshold of caregiving hours by entering three independent caregiving-hours-related variables into the probit model as recently proposed by Van Houtzen and colleagues [8]: (1) a continuous variable representing caregiving hours, CG; (2) a dummy variable that indicated whether caregiving hours exceeded a threshold, CG^; and (3) an interaction term between caregiving hours and the threshold dummy variable, CG*CG^. Each of the three estimated coefficients on these caregiving variables reflects the incremental change in the likelihood of LFP because of a unit increase in caregiving hours, an abrupt discontinuity in the relationship between caregiving and LFP at the caregiving threshold and potential change to the incremental effect of caregiving on LFP when caregiving hours exceed the threshold. In an iterative selection procedure, we tested all potential thresholds of caregiving hours (between 0–140 with increments of 1–10 h depending on the availability of observations) to identify the threshold that maximized the likelihood of the corresponding probit model. Using this threshold in the probit model, we conducted two joint Wald tests: (1) first, we tested the null hypothesis that the coefficients of the three caregiving variables were all zero. Rejecting this hypothesis confirmed the significance of an overall association between LFP and the set of caregiving variables; (2) next, we tested if the coefficient of the caregiving threshold dummy variable, CG^, and that of the interaction term, CG*CG^, were jointly zero to assess whether there was a significant change in the association between caregiving and LFP once caregiving hours were at or beyond the threshold.

We also performed an instrumental variables analysis to correct for potential endogeneity of caregiving hours [34]. For both females and males, this technique was employed to address two statistical challenges: first, the potential for an inverse relationship between LFP and caregiving hours; second, the presence of unmeasured confounders (such as low career ambitious or a preference for family caregiving). We used three instrumental variables for weekly caregiving hours, including the number of grandchildren aged below 16; whether the husband’s father was widowed; and whether the wife’s father was widowed. These instruments are established in the international literature [2,11,28,34] and meet the required assumptions [35]: (1) as Chinese adults are legally obligated to support and take care of their elderly parents [36] and likely to take on the parental role of their grandchildren [7], it is therefore reasonable to assume that individuals tasked with heavy unpaid caregiving duties at home (captured by the three instruments) would tend to allocate more time to unpaid caregiving in order to fulfil their obligations; (2) the three instruments are not correlated with LFP as prior literature based in developing countries [37,38,39], especially in Asian countries [5,40,41,42], has identified the determinants of LFP to be education attainment and external factors such as urban location. 

Using these instruments, we performed a Limited-Information Maximum Likelihood (LIML) procedure [43,44]. This method was chosen over the more commonly used procedure—Two-Stage Least-Squares—because it results in less bias to the estimates when the IVs are weakly associated with the endogenous variable [45]. In the first equation, the three instruments were entered into a linear regression to predict caregiving hours, controlling for all covariates. In the second equation, caregiving hours were used in the threshold-adjusted probit model to predict LFP, after adjustment for covariates. We used the same caregiving threshold that had been previously identified in the one-step probit regression analysis. We assessed whether the predictions from a model treating the caregiving hours variable as exogenous differed significantly from a model where it was treated as endogenous using two Sargan-Hansen statistics [46,47]. We tested the validity of our instruments with tests of under-identification (using the Anderson canon. corr. LM statistic), over-identification (i.e. the Sargan-Hansen test of over-identifying restrictions) and weak identification (i.e. the Cragg–Donald Wald F-statistic in the first equation). The one-step probit model (without the use of instruments) was deemed more appropriate when we failed to reject the exogeneity of caregiving hours.

Using either the LIML model or the one-step probit model (whichever was deemed to be more appropriate), we predicted the probability of LFP separately for a Chinese men and women with reference-level characteristics who spent between 0 and 140 h a week on unpaid caregiving. We defined Chinese women with reference-level characteristics to be 50 years of age, married, with middle school education, living in a rural community with 4 household members, did not have work-limiting health conditions, and whose spouse earned 1,466 RMB per month. A Chinese man with reference-level characteristics shared the same characteristics except for being 52 years of age with a spouse and monthly income of 442 RMB. 

### 2.5. Sensitivity Analysis

For women and men separately, we conducted three sets of sensitivity analysis. The first set of analysis entails the estimation of four simpler models of unpaid caregiving hours and LFP following the specifications used in the prior literature [2,9,10,11,13,14,15,16]. The first model regarded caregiving as a dummy variable (denoting caregivers vs. noncaregivers) considering neither kinks nor discontinuities; the other three models excluded the possibility of a discontinuity or a kink or both. A pairwise likelihood ratio test was performed to compare each simpler model with our model to establish the statistical value of accounting for the presence of both kinks and discontinuities in the estimation of the relationship between unpaid caregiving hours and LFP. 

Next, we undertook subgroup analyses stratified by education attainment (below middle school vs. middle school or above), types of Hukou (urban vs. rural), household income (below the median income vs. equal to or above the median income) and regions of Hukou (in East, Central and West China; and in a Tier 2 or above vs. a below Tier 2 city). Using the weekly caregiving threshold yielded in the primary analysis, we repeated the analysis on each subgroup to verify the robustness of our primary findings. 

Last, separate analyses were conducted to assess the LFP relationship with hours of grandchild care and with hours of eldercare (provided to parents and/or parents-in-law), respectively. For each type of caregiving and for women and men separately, we performed the instrumental variables analysis using the LIML procedure; a one-step probit analysis without the use of instruments was undertaken if endogeneity of caregiving hours was rejected. For the grandchild care analysis, three instruments were used: the presence of grandchildren aged below 16 (yes/no); the number of these young grandchildren; and the number of kindergartens in the community. For the eldercare analysis, another three instruments were used: the number of parents and parents-in-law that were alive; whether one of parents or parents-in-law was in poor health (yes/no); and the presence of eldercare facilities in the community (including publicly financed nursing homes, organizations for helping the elderly, elderly activity centers, home-based eldercare centers and elderly primary care centers) [26]. A new caregiving threshold was located in each instance using the same iterative procedure. All analyses were conducted using Stata/SE 15.0 (StataCorp LLC, College Station, TX, USA).

## 3. Results

### 3.1. Sample Characteristics

Table 1 reports the baseline characteristics of respondents by LFP status and by gender. Among a total of 3718 (64.2%) labor force participants, there were 681 labor force participating women and 1,706 men. Compared to men, women were younger (mean age = 48.8 vs. 51.6 years, *p*-value < 0.01), living in smaller households (mean size = 3.5 vs. 3.8 persons, *p*-value < 0.01) and had a spouse with higher monthly income (mean income = 1,822.8 RMB vs. 502.2 RMB, *p*-value < 0.01). Labor force participating women were also less likely to be married (94% vs. 97%, *p*-value < 0.01), having completed middle school (58% vs. 66%, *p*-value < 0.01) and living in rural areas (36% vs. 44%, *p*-value < 0.01). The hours of unpaid caregiving per week did not differ between labor force participating women and men.

We observed similar differences between gender groups among non-participants (*N* = 1,331, 35.8%), whereas women were also younger (mean age = 50.6 vs. 54.4 years, *p*-value < 0.01), with lower education (percentage of middle school graduates = 47% vs. 59%, *p*-value < 0.01) and had a spouse with higher monthly income (mean income = 1,149.7 RMB vs. 259.0 RMB, *p*-value < 0.01). Hours of unpaid caregiving, household size, marital status and urban/rural residence did not differ between women and men who were non-participants. 

Regardless of LFP status, overall, women in our sample were younger (mean age = 49.7 vs. 52.3 years, *p*-value < 0.01), with lower education (percentage of middle school graduates = 52% vs. 64%, *p*-value < 0.01) and had a spouse with higher monthly income (mean income = 1,465.8 RMB vs. 442.0 RMB, *p*-value < 0.01). Women were also less likely to reside in rural areas (37% vs. 44%, *p*-value < 0.01) or to report work-limiting health conditions (10% vs. 13%, *p*-value < 0.01). Furthermore, women provided 7 more hours of unpaid caregiving per week on average (mean caregiving hours = 18 vs. 11, *p*-value < 0.01).

### 3.2. Endogeneity of Unpaid Caregiving Hours

For both women and men, the three instruments passed the over-identifying restriction test (both *p*-values > 0.1; see Appendix A), but we failed to reject under-identification on both occasions (both *p*-values > 0.1). Furthermore, for both gender groups, the three instruments were weak (Cragg–Donald Wald F-statistic = 0.803 and 1.198 for women and men, respectively). Nevertheless, use of the three instruments was still deemed appropriate as the Limited-Information Maximum Likelihood (LIML) estimator tended to be robust to weak instruments [45]. Using these instruments, we failed to reject the exogeneity of unpaid caregiving hours for both women and men (both *p*-values > 0.1). Consequently, the use of an instrumental variables approach was ruled out in both cases, and accordingly, we only present results of the one-stage probit analysis below (Table 2). Two sets of instrumental variables analyses using a two-stage least-square and a LIML procedure yielded largely similar results (Appendix A). 

### 3.3. Association between Unpaid Caregiving Hours and LFP

For women, testing various caregiving thresholds between 1–140 hrs/w yielded probit models with log-likelihood values ranging from –820.8 (corresponding to a caregiving threshold of 12 hrs/w) to a high of –817.2 (corresponding to a caregiving threshold of eight hrs/w). Hence, the threshold of unpaid caregiving was identified to be eight hrs/w for women. When compared to women who provided less than eight hours of caregiving per week (Appendix B), those offering at least eight hours of caregiving per week were slightly older (mean age = 50.5 years vs. 49.4 years, *p*-value < 0.01) and less likely to be married (92% vs. 96%, *p*-value < 0.01); having graduated from college (2% vs. 5%, *p*-value < 0.05); and having work-limiting health conditions (7% vs. 12%, *p*-value < 0.05). They were also living in larger households (4.1 vs. 3.4 persons, *p*-value < 0.01) and less likely to be employed (percentage of labor force participants = 35% vs. 53%, *p*-value < 0.01).

Using the eight-hour threshold, we found that before eight hours, each unpaid caregiving hour was significantly associated with a higher likelihood of LFP (probit coefficient = 0.0804, 95% CIs: 0.0123 to 0.149, *p*-value < 0.05), which corresponds to an increase of 0.0257 (95% CIs: 0.00394 to 0.0474) in the marginal probability of LFP. There was no evidence of any significant abrupt change in LFP at the threshold (probit coefficient = –0.0840, 95% CIs: –0.313 to 0.144, *p*-value > 0.1). After the threshold, we observed a significant decrease of 0.0271 (95% CIs: –0.0488 to –0.0054) in the slope of the marginal probability of LFP for each additional unpaid caregiving hour above the caregiving threshold (probit coefficient of the interaction = –0.0847, 95% CIs: –0.153 to –0.0165, *p*-value < 0.05). These findings imply that each unpaid caregiving hour for women beyond eight hrs/w was associated with a reduction of 0.0014 in the marginal probability of LFP. Overall, there was strong evidence of a significant association between unpaid caregiving hours and LFP among women (joint *p*-value of three caregiving hours variables < 0.001), and that there was a differential association between caregiving hours and LFP below and above the caregiving threshold of eight hrs/w (joint *p*-value of the threshold and interaction = 0.028). 

Other significant correlates of higher LFP among women were: younger age (for a one-year increase in age: marginal probability = –0.0312, 95% CIs: –0.0380 to −0.0244, *p*-value < 0.01); unmarried (married vs. unmarried: marginal probability = –0.134, 95% CIs: −0.237 to –0.0316, *p*-value < 0.05); having completed high school (marginal probability = 0.0743, 95% CIs: 0.0122 to 0.136, *p*-value < 0.05) or college and above (marginal probability = 0.332, 95% CIs: 0.197 to 0.467, *p*-value < 0.01); reporting work-limiting health issues (marginal probability = 0.539, 95% CIs: 0.441 to 0.637, *p*-value < 0.01); and a higher monthly spousal income (logged income: marginal probability = 0.0173, 95% CIs: 0.0113 to 0.0232, *p*-value < 0.01).

For men, the log-likelihood function of the probit model ranged from –1089 (corresponding to a caregiving threshold of 1 h per week) to –1087 (corresponding to a caregiving threshold of 72 hrs/w). Hence, the caregiving threshold was estimated to occur at 72 h of caregiving per week. Compared to men who provided less than 72 h of caregiving per week (Appendix B), those tasked with heavier caregiving duties were 2-years older on average (mean age = 54.2 vs. 52.2 years, *p*-value < 0.01), more likely to report work-limiting health conditions (21% vs. 13%, *p*-value < 0.01), and living in larger households (4.6 vs. 3.7 persons, *p*-value < 0.01). With regard to LFP status, men providing at least 72 h of caregiving per week were less likely to be employed (percentage of labor force participants = 67% vs. 76%, *p*-value < 0.01).

Using the 72-h caregiving threshed, we found that before 72 h, each caregiving hour was significantly associated with lower LFP (probit coefficient = –0.00442, 95% CIs: –0.00822 to –0.000612, *p*-value < 0.05), such that an hourly increment in caregiving reduced the probability of LFP by 0.00119 (95% CIs: –0.00222 to –0.000169) at the margin. There was neither a significant change in LFP at the caregiving threshold (probit coefficient = 1.262, 95% CIs: –0.178 to 2.702, *p*-value > 0.1), nor for the relationship between LFP and incremental changes in caregiving hours below or above that threshold. Specifically, the marginal probability of LFP fell continuously with more caregiving hours in the pre- and post-threshold periods (probit coefficient of interaction = –0.00852, 95% CIs: –0.0212 to 0.00418, *p*-value > 0.1). In conclusion, we found strong evidence of an overall association between caregiving hours and LFP for men (joint *p*-value of three caregiving hours variables = 0.0120), but this association did not depend on the caregiving threshold (joint *p*-value of the threshold and interaction = 0.160). 

Other significant correlates of higher LFP among men were younger age (for a one-year increase in age: marginal probability = –0.0180, 95% CIs: –0.0215 to –0.0145, *p*-value < 0.01); married (vs. unmarried: marginal probability = 0.108, 95% CIs: 0.0351 to 0.181, *p*-value < 0.01); having completed high school (marginal probability = 0.0564, 95% CIs: 0.0111 to 0.1017, *p*-value < 0.05); urban residence (marginal probability = 0.0793, 95% CIs: 0.0447 to 0.114, *p*-value < 0.01); reporting work-limiting health conditions (marginal probability = 0.434, 95% CIs: 0.333 to 0.535, *p*-value < 0.01); and a higher monthly spousal income (logged income: marginal probability = 0.0132, 95% CIs: 0.00726 to 0.0191, *p*-value < 0.01). 

**Figure 1:**
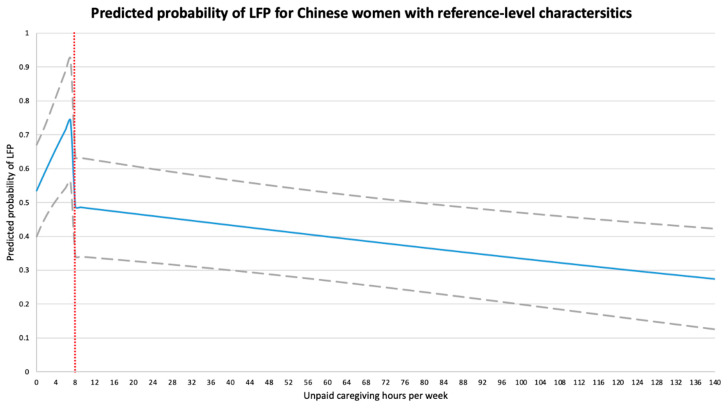
Predicted probability of labor force participation based on the one-stage probit model for Chinese women with reference-level characteristics. We defined Chinese women with reference-level characteristics to be 50 years of age, married, with middle school education, living in a rural community with 4 household members, did not have work-limiting health conditions, and whose spouse earned 1466 RMB per month. Grey dashed lines represent the 95% confidence intervals for the predicted probability of labor force participation. LFP, labor force participation.

In Figure 1, we report the predicted probability of LFP and its 95% CIs with different unpaid caregiving hours for Chinese women with reference-level characteristics. When women were not caregivers, their probability of LFP was 0.535 (95% CIs: 0.400 to 0.670), but with unpaid caregiving, the probability of LFP would initially grow to 0.743 (95% CIs: 0.562 to 0.924) at 7 caregiving hrs/w. At the caregiving threshold of eight hours, there was an estimated, though not statistically significant, discontinuity as the probability of LFP fell abruptly to 0.488 (95% CIs: 0.342 to 0.634), and with caregiving hours beyond the caregiving threshold the probability of LFP fell continuously from 0.488 to just 0.274 (95% CIs: 0.126 to 0.423) once she reached 140 caregiving hours a week.

**Figure 2:**
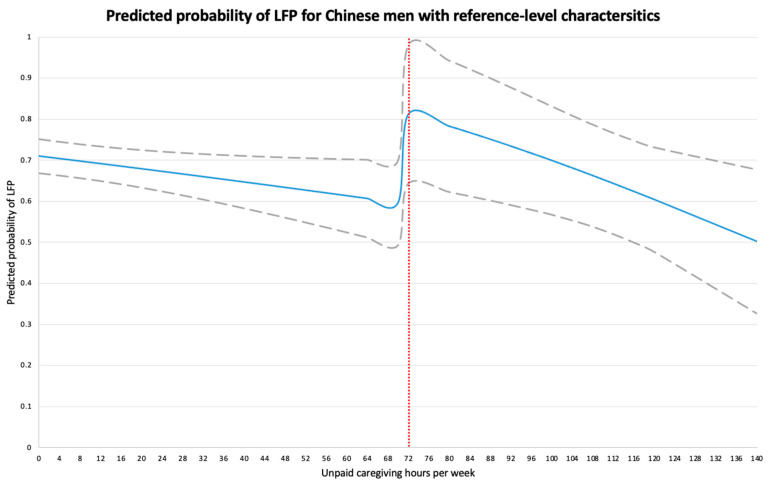
Predicted probability of labor force participation based on the one-stage probit model for Chinese men with reference-level characteristics. We defined Chinese male with reference-level characteristics to be 52 years of age, married, with middle school education, living in a rural community with 4 household members, did not have work-limiting health conditions, and whose spouse earned 442 RMB per month. Grey dashed lines represent 95% confidence intervals for the predicted probability of labor force participation. LFP, labor force participation.

In Figure 2, we report the predicted probability of LFP and 95% CIs for reference-level Chinese men with different weekly hours of unpaid caregiving. We observed a steady decline in their probability of LFP from 0.710 (95% CIs: 0.669 to 0.752) to 0.597 (95% CIs: 0.493 to 0.700) as their unpaid caregiving hrs/w increased from 0 to 71 h. At the caregiving threshold of 72 h, the probability reached a high of 0.811 (95% CIs: 0.643 to 0.980), but thereafter the probability fell with more caregiving hours, ultimately reaching a low of 0.502 (95% CIs: 0.326 to 0.677) at 140 h of unpaid caregiving a week.

### 3.4. Sensitivity Analysis

We report estimation results for the four simpler models in Table 3. For women, we found strong statistical evidence that omitting considerations of either kinks or discontinuities or both would greatly reduce the performance of the model (all *p*-values of likelihood ratio tests < 0.05). For men, the results implied that while our model exceeded the performance of the model that regarded caregiving as a dummy variable and the discontinuity-only model (both *p*-values of likelihood ratio test > 0.05), it was comparable to the kink-only model (*p*-value < 0.1) and to the model that accounted for neither kinks nor discontinuities (*p*-value > 0.1).

Results of subgroup analyses stratified by the type of Hukou status, educational level, household income and Hukou region for Chinese women are reported in Table 4. We found that for women who either had urban Hukou status or at least middle school education or household income that equaled to or exceeded the median level or having registered their Hukou in a city that was below Tier 2, the relationship between their unpaid caregiving hours and LFP generally followed the pattern revealed in our primary analysis; that is, before eight hours, LFP tended to increase with more unpaid caregiving hours but any additional caregiving hours thereafter reduced LFP. For women with rural Hukou status, the pre-threshold increasing association between caregiving hours and LFP diminished (*p*-value of the caregiving continuous variable > 0.1), and for those with household income below the median level or having their Hukou registered in either the West Chinese region or in a city that was ranked Tier 2 or above, there was an absence of association between unpaid caregiving hours and LFP (joint *p*-value of three caregiving hours variables > 0.05).

We report results of the same subgroup analyses on Chinese men in Table 5. We did not identify any association between unpaid caregiving hours and LFP for men with urban Hukou status, had at least middle school education, came from a household with income below the median level, or had their Hukou registered in the East or West Chinese regions (all *p*-values of the joint significance of the three caregiving hours variables > 0.1). For men with rural Hukou status or did not complete middle school or had their Hukou registered in the Central Chinese region, their LFP decreased continuously with more unpaid caregiving hours without the effects of any discontinuities or kinks at the 72-h caregiving threshold. For men whose household income was at least at the median level, their LFP was initially unrelated to more unpaid caregiving hours before the 72-h threshold (*p*-value of the caregiving continuous variable > 0.1); at the threshold, there was an increase of 0.969 in the marginal probability of LFP (*p*-value < 0.05) and LFP started to decrease with more caregiving hours thereafter (joint *p*-value of the threshold and interaction < 0.05). 

The relationship between LFP and hours of grandchild care was assessed for women and men. We failed to reject the exogeneity of hours of grandchild care for both genders (Appendix C, Table A3), therefore results of a one-step probit model are reported (Table 6). For women, our analysis yielded 4-hrs/w as an important threshold of grandchild care. Although the three caregiving hours variables were individually insignificant, we did find the presence of an overall negative association between women’s LFP and hours of grandchild care (*p*-value<0.01). For men, we identified a negative association between LFP and hours of grandchild care (*p*-value < 0.01) and a threshold of grandchild care at 72-hrs/w. Before 72 h, each hour of grandchild care was significantly associated with a 0.00199 decrease in men’s marginal probability of LFP (*p*-value < 0.01). There was an abrupt but statistically insignificant rise of LFP at the 72-h threshold, and additional grandchild care hours beyond the threshold continued to lower men’s marginal probability of LFP by 0.00199. Hence, the threshold effect among men was insignificant (*p*-value > 0.05).

We also examined the relationship between LFP and hours of eldercare provided to parents and/or parents-in-law. For both women and men, we found strong evidence to reject the exogeneity of eldercare hours, and thereby corroborated the use of the three instrumental variables (Table 6; Appendix C, Table A4). For women, 7-h of eldercare per week was identified as a threshold. Before 7 h, each eldercare hour was significantly associated with a 0.365 increase in women’s marginal probability of LFP (*p*-value < 0.05). Although there was an absence of discontinuity at the 7-h threshold, each eldercare hour thereafter was associated with lower LFP by reducing the marginal probability of LFP by 0.001 (*p*-value < 0.05), which gave rise to a significant threshold effect (*p*-value < 0.05). For men, the threshold of eldercare occurred at 70-hrs/w. Before 70 h, each eldercare hour was associated with higher LFP that did not reach statistical significance (*p*-value > 0.05). Neither the discontinuity nor the kink was individually significant (both *p*-values > 0.1); however, we did identify a significant threshold effect that might indicate a net positive change in LFP once men’s eldercare hours reached or exceeded the 70-h threshold (*p*-value < 0.05). An overall association between men’s LFP and eldercare hours was also significant (*p*-value < 0.05). 

## 4. Discussion

In this population-based cross-sectional study, we used data from the CHARLS baseline survey to explore the relationship between weekly unpaid caregiving hours and LFP among Chinese women and men. Three major findings emerged from our analysis: first, LFP was significantly associated with caregiving for both gender groups. Second, although we did identify a caregiving threshold (72 hrs/w) for men, their LFP was generally inversely related to caregiving without any kinks or discontinuities. Third, we identified a statistically significant kink in the relationship among women whereby their probability of LFP was initially positively associated with caregiving until it reached a caregiving threshold of eight hrs/w after which the probability of LFP fell continuously with more caregiving hours. 

### 4.1. In Contrast to Prior Literature

To the best of our knowledge, our work is the first empirical analysis that simultaneously assessed both kinks and discontinuities in the relationship between caregiving and labor force participation status. Furthermore, we established the statistical value of considering both kinks and discontinuities through examining four simpler models all of which had poorer performance. A recently proposed theoretical model suggests LFP would be inversely related to caregiving intensity with one discontinuity in this relationship [8]. Our analysis attests to this non-linearity, but there are other findings that are not in line with those theoretical predictions: first, among both women and men, we did not identify any statistically significant discontinuity (both *p*-values > 0.1) in their respective LFP relationships; second, the relationship between caregiving intensity and LFP for women was not consistently negative; LFP first grew with caregiving hours, reaching a peak at 7-hrs/w of caregiving, and then falling from the caregiving threshold of 8-hrs/w. While the first discrepancy is largely attributed to the structure of our data, the second warrants further consideration. It is plausible that our results imply that when confronted with the double burdens of paid work and caregiving responsibilities, Chinese women are inclined to combine labor work with a moderate amount of caregiving; it is only when these caregiving duties become more time-consuming that they tend to withdraw from the labor market. Hence, our results suggest that while caregiving might exert an adverse impact on the employment opportunities of Chinese men, Chinese women are more likely to balance their work and caregiving activities, at least until their intensity of caregiving reached the caregiving threshold. These findings are unique in the international literature and contrast with prior studies that suggest caregivers are generally more likely to withdraw from the labor market given more intensive caregiving [2,9,11,15,48,49]. We add to the literature by identifying a segment of the LFP relationship with caregiving intensity that is not exclusively decreasing, at least for women who provide unpaid care up until the threshold.

Regarding potentially significant thresholds of weekly unpaid caregiving hours, prior studies have explored four candidates (including 0, 5, 10, 15 and 20 h), but these were either chosen conveniently in increments of 5 h or were loosely based on prior findings [12]. For the first time, we were able to locate two caregiving thresholds—for women and men separately—that were statistically grounded and verified the effects of these thresholds by joint hypothesis testing. There has been no Asian-based study that has examined a caregiving threshold, so our study represents an advancement in that regard [5,41,50,51,52]. 

Another novelty of our analysis was to simultaneously deal with the potential endogeneity of unpaid caregiving hours and locate empirically a caregiving threshold. Although in the theoretical model proposed by Van Houtven et al [8] caregiving hours are considered exogenous, we used instrumental variables to statistically rule out the potential for inverse causality and unmeasured confounding while embedding a maximized likelihood-based procedure to identify a significant caregiving threshold. The popularity of instrumental variables is well established in the health economics literature and at least five studies have applied this technique to understand the causal role of unpaid caregiving on labor market outcomes [2,11,28,34,50]. However, there is a paucity of work that jointly use instrumental variables and a selective procedure to detect thresholds [53]. Our paper demonstrates that it is feasible to combine such methods and we hope that it may encourage others to replicate such methods when assessing a complex causal relationship involving potential kinks and/or discontinuities.

Our work identified a significant positive association between LFP and having work-limiting health conditions. Specifically, women and men with work-limiting health conditions in our sample were associated with 0.539 and 0.434 increase in the marginal probability of LFP, respectively. While this positive association may seem counter-intuitive and contrast with international literature [54], it is imperative to note that our study sample comprises nonfarming, working-age Chinese (aged 45–65 years) who face retirement, if they are not already retired [25]. So, for our study participants the decision they face is whether to exit the labor market or to continue working until they reach their mandatory age of retirement. In this way, our results imply that having a work-limiting health condition might act as a proxy for low levels of accumulated wealth whereby healthy individuals (with higher accumulative wealth) tend to retire earlier than those with work-limiting health conditions. The latter tend to have lower wealth and greater financial insecurity are likely to work towards their mandatory retirement age in order to enable them to live more independently in older age. Future studies with data on common measures of accumulated wealth, such as net worth, home ownership and total assets, need to confirm the proxy role of having work-limiting health conditions on low wealth [55]. As China is ready to raise the mandatory retirement age [56], it is important to monitor the trend of employment among those with work-limiting health conditions to allow for the design of welfare programs that aid the well-being of those individuals.

### 4.2. Policy Implications

Our findings have important implications for policy decision makers. Within China’s institutional and cultural context, unpaid caregiving by family members is expected to continue to be the predominant source of care in future years [36]. As such, policy makers need to be well-informed about the trade-off between increased unpaid caregiving and erosions in labor market participation. By designing interventions that help unpaid caregivers better balance their caregiving commitment and labor market responsibility, there is potential to advance both sets of activities. This could be accomplished in many ways, including but not limited to more flexible work hours, paid leave for caregiving and care allowances. Depending on the type of caregiving, targeted interventions could be implemented to benefit childcare and eldercare providers. These include publicly funded daycare, expanded insurance coverage for children with complex medical needs, government assistance for long-term care accommodations for seniors and establishments of community-based eldercare facilities. Such programs have already been implemented successfully in some western countries with proven effectiveness in alleviating the burden of caregiving and supporting caregivers to balance paid work and caregiving obligations [57]. Moreover, as we found Chinese women and men react differently when confronted with the double burden of caregiving and employment, policies need to reflect this gendered difference. Specifically, family-friendly policies need to target women who combine work and caregiving in order to enhance their ability to actively take part in both unpaid home care and labor activities, with the goal of advancing their well-being. There is evidence from some western countries that such family-friendly policies targeting women are promising tools to promote a higher economic activity of women in addition to improving the work-family balance for both gender groups [58]. Furthermore, welfare programs that support male employees with family caregiving duties are nearly non-existent in China [59]. As we found Chinese middle-aged men, especially those providing care to grandchildren, tend to withdraw from work given increased caregiving tasks, efforts must be made to design interventions to aid men who engage in both paid work and unpaid caregiving. 

### 4.3. Limitations

Our study has a number of limitations that are common in observational studies using cross-sectional survey data. First, accuracy of these data relied entirely on self-reporting by participants. However, the CHARLS is a nationally representative survey with rigorous sampling procedures and well-established survey instruments [26] which should lead to reliable responses among participants. Second, our study is uniquely situated in China, which impedes our ability to generalize the findings to other countries. However, we do believe that the increasing burden of unpaid caregiving is a shared concern worldwide [1,2], and the results from our analysis provide insights that are applicable to an international context. Furthermore, the significance of our study is that for the first time, a complex relationship between LFP and unpaid caregiving that entails potential kinks and discontinuities has been empirically examined. In this way, our work provides important statistical insights and paves the way forward for others to replicate this analysis using international data. We were unable to account for the effect of policy reforms that occurred after 2011, including the replacement of the one-child policy with a two-child policy [60], the expansion of welfare program for disabled persons [61] and the extension of maternity/paternal leaves in some regions [59]. In particular, starting in Guangzhou, China has launched a Hukou reform in May 2010 by introducing the Unified Residence Hukou as a third class of Hukou beyond Agricultural Hukou and Non-agricultural Hukou [62]. Due to the nascent status of this new Hukou class, only 0.6% (*N* = 107) of all CHARLS baseline survey participants reported to have this class of Hukou. Hence, future studies need to assess the impact of this Hukou reform in the analysis of caregiving and labor engagement. Furthermore, we were also unable to assess a causal relationship between unpaid caregiving and LFP due to the cross-sectional nature of our data as well as the possibility of having multiple meaningful caregiving hours thresholds due to our relatively small sample size [8]. Hence, future study with access to larger and more recent longitudinal data need to re-visit this topic in order to examine the causal role of unpaid caregiving on various labor market outcomes in the current era. Finally, we were unable to account for the potential disparity amongst individuals’ region of Hukou registration, the region where unpaid caregiving activities took place and the region of labor force participation and thereby did not identify subgroups of workers (such as rural migrant workers) whose place of caregiving and working might differed [63]. Future studies with those data will provide additional insights on how middle-aged Chinese adults find the balance between caregiving and working.

## 5. Conclusions

This study offers important empirical insights regarding the complex relationship between the intensity of unpaid caregiving and labor force participation among Chinese women and men. The findings help inform both health and social care policy decision making as well as labor force policy in the face of an aging of the population. Policies that assist unpaid caregivers to maintain balance in their caregiving and labor market activities are of universal importance. Moreover, there are opportunities to extend the methodology to other labor market outcomes that may be impacted by unpaid caregiving, such as hours of work and hourly wages.

## Figures and Tables

**Table 1:** Compare the characteristics of survey respondents by labor force participation status and by gender.

Characteristics	Labor Force Participants			Non-Participants			Total		
	(*N* = 2387, 64.2%)			(*N* = 1331, 35.8%)			(*N* = 3718)		
	Women	Men	*p*-Value	Women	Men	*p*-Value	Women	Men	*p*-Value
N (%)	681 (29)	1706 (71)		769 (58)	562 (42)		1450 (39)	2268 (61)	
Unpaid caregiving, hr/wk, Mean (SD)	11 (24.3)	9 (23.6)	>0.1	24 (38.5)	15 (31.6)	>0.1	18 (33.3)	11 (25.9)	***
Age, yr, Mean (SD)	48.8 (3.0)	51.6 (4.5)	***	50.6 (3.2)	54.4 (4.6)	***	49.7 (3.2)	52.3 (4.7)	***
Married, N (%)	643 (94)	1651 (97)	***	729 (95)	519 (92)	*	1372 (95)	2170 (96)	>0.1
Education, N (%)			***			***			***
Illiterate/elementary school	283 (42)	578 (34)		406 (53)	231 (41)		689 (48)	809 (36)	
Middle school	187 (27)	597 (35)		200 (26)	194 (35)		387 (27)	791 (35)	
High school	167 (25)	429 (25)		153 (20)	108 (19)		320 (22)	537 (24)	
College and above	44 (6)	102 (6)		10 (1)	29 (5)		54 (4)	131 (6)	
Urban residence, N (%)	439 (64)	913 (54)	***	479 (62)	367 (65)	>0.1	918 (63)	1280 (56)	***
Having work-limiting health conditions, N (%)	136 (20)	300 (18)	>0.1	10 (1)	5 (1)	>0.1	146 (10)	305 (13)	***
Household size, Mean (SD)	3.5 (1.3)	3.8 (1.6)	***	3.7 (1.6)	3.6 (1.7)	*	3.6 (1.5)	3.8 (1.6)	>0.1
Monthly income of spouse, RMB, Mean (SD)	1822.8	502.2	***	1149.7	259	***	1465.8	442	***
	-3028.8	-1427.6		-2148.4	-976.8		-2620	-1334.1	

Continuous variables were compared using the 2-sample t-test (mean). Categorical variables were compared using the Chi-square test. Abbreviations: SD, standard deviation; yr, year; hr, hour; wk, week; RMB, Renminbi (Chinese Yuan). * *p*-value<0.1, ** *p*-value<0.05, *** *p-*value < 0.01.

**Table 2:** Association of weekly unpaid caregiving hours and labor force participation estimated by the one-stage probit model.

	Women (Threshold = Eight Hours)		Men (Threshold = 72 Hours)	
Variables	Probit Coefficient (95% CI)	Marginal Probability (95% CI)	Probit Coefficient (95% CI)	Marginal Probability (95% CI)
Caregiving hours before the threshold, per hour, CG	0.0804 ** (0.0123, 0.149)	0.0257 ** (0.00394, 0.0474)	−0.00442 ** (−0.00822, −0.000612)	−0.00119 ** (−0.00222, −0.000169)
Discontinuity at the threshold of caregiving, CG^	−0.0840 (−0.313, 0.144)	−0.0269 (−0.100, 0.0462)	1.262 (−0.178, 2.702)	0.341 (−0.0474, 0.730)
Interaction between threshold and caregiving, CG*CG^	–0.0847 ** (−0.153, −0.0165)	–0.0271 ** (−0.0488, −0.0054)	–0.00852 (−0.0212, 0.00418)	–0.00230 (−0.00573, 0.00113)
Caregiving hours after the threshold, per hour †	−0.0043 **	−0.0014 **	−0.0129	−0.00349
Age, per one-year increase	−0.0975 *** (−0.120, −0.0746)	−0.0312 *** (−0.0380, −0.0244)	−0.0665 *** (−0.0803, −0.0526)	−0.0180 *** (–0.0215, –0.0145)
Married	−0.420 ** (−0.743, −0.0966)	−0.134 ** (–0.237, –0.0316)	0.400 *** (0.128, 0.673)	0.108 *** (0.0351, 0.181)
Middle school	0.0675 (−0.113, 0.248)	0.0216 (−0.0360, 0.0792)	0.0338 (−0.112, 0.179)	0.00915 (−0.0302, 0.0485)
High school	0.232 ** (0.0370, 0.427)	0.0743 ** (0.0122 0.136)	0.208 ** (0.0403, 0.376)	0.0564 ** (0.0111, 0.102)
College and above	1.037 *** (0.604, 1.470)	0.3319 *** (0.197, 0.467)	0.186 (−0.0978, 0.469)	0.0502 (−0.0264, 0.127)
Urban residence	0.0755 (−0.0870, 0.238)	0.0242 (−0.0278, 0.0761)	0.293 *** (0.163, 0.423)	0.0793 *** (0.0447, 0.114)
Having work-limiting health conditions	1.685 *** (1.349, 2.021)	0.539 *** (0.441, 0.637)	1.604 *** (1.222, 1.986)	0.434 *** (0.333, 0.535)
Household size	–0.0239 (–0.0747, 0.0269)	–0.00764 (–0.0238, 0.00857)	0.0275 (–0.0113, 0.0663)	0.00744 (–0.00304, 0.0179)
Monthly spousal income (log-transformed)	0.0540 *** (0.0348, 0.0733)	0.0173 *** (0.0113, 0.0232)	0.0487 *** (0.0266, 0.0707)	0.0132 *** (0.00726, 0.0191)

† The probit coefficient representing the association between labor force participation and caregiving hours after the threshold was calculated by summing the coefficients of CG and CG*CG^. Its *p*-value represents the joint significance of CG and CG*CG^. ** *p*-value < 0.05, *** *p*-value < 0.01. CG, caregiving; CI, confidence interval.

**Table 3:** Results of four simpler models for estimating the relationship between unpaid caregiving hours and LFP.

Variables	CG as a Dummy Variable	CG as a Continuous Variable	Discontinuity-Only	Kink-Only
**Women**				
Caregivers status	−0.0608 * (0.0242)	--	--	--
Caregiving hours before the threshold, per hour	--	−0.00176 *** (0.000376)	0.0875 * (0.0456)	−0.00179 (0.00416)
Discontinuity at the threshold of caregiving	--	--	−0.185 *** (0.0475)	--
Interaction between threshold and caregiving (kink)	--	--	--	2.92 × 10^−5^ (0.00452)
Pairwise likelihood ratio test (vs. the original model)	***	**	***	***
**Men**				
Caregivers status	−0.00119 * (0.000523)	--	--	--
Caregiving hours before the threshold, per hour	--	−0.0187 (0.0183)	−0.000846 *** (0.000315)	−0.000803 * (0.000486)
Discontinuity at the threshold of caregiving	--	--	−0.0411 (0.0449)	--
Interaction between threshold and caregiving (kink)	--	--	--	−0.000153 (0.00132)
Pairwise likelihood ratio test (vs. the original model)	***	>0.1	***	*

We report the marginal effect point estimate and the standard error. * *p*-value < 0.1, *** *p*-value < 0.01.

**Table 4:** Subgroup analyses on Chinese women assuming a weekly caregiving threshold of eight hours.

	Hukou Status		Educational Status		Household Income		Hukou Region (Economic Macro-Regions)			Hukou Region (City Tiers)	
Variables	Urban (*N* = 544)	Rural (*N* = 902)	Below Middle School (*N* = 689)	Middle School or above (*N* = 762)	Below the Median (*N* = 649)	Equal or above the Median (*N* = 796)	East (*N* = 609)	Central (*N* = 480)	West (*N* = 361)	Tier 2 or above (*N* = 465)	Below Tier 2 (*N* = 985)
Caregiving hours before the threshold, per hour	0.0275 * (0.0147)	0.0257 (0.0264)	0.0293 * (0.0162)	0.0300 ** (0.0146)	0.00776 (0.0179)	0.0355 ** (0.0142)	0.0206 (0.0142)	0.00582 (0.0238)	0.0237 (0.0284)	0.0102 (0.0162)	0.0358 ** (0.0151)
Discontinuity at the threshold of caregiving	−0.0445 (0.0637)	−0.0116 (0.0468)	0.0113 (0.0543)	−0.0507 (0.0524)	−0.0554 (0.0535)	0.0354 (0.0515)	−0.0971* (0.0570)	−0.0534 (0.0680)	0.0863 (0.0671)	0.0259 (0.0672)	−0.0719 (0.0449)
Interaction between threshold and caregiving	−0.0289 ** (0.0147)	−0.0272 * (0.0164)	−0.0314 * (0.0162)	−0.0309 ** (0.0147)	−0.00842 (0.0179)	−0.0377 *** (0.0142)	−0.0211 (0.0142)	−0.00743 (0.0238)	−0.0264 (0.0285)	−0.0122 (0.0162)	−0.0367 ** (0.0151)
Caregiving hours after the threshold, per hour †	−0.0014 *	−0.0015	−0.0021	−0.0009 **	−0.00066	−0.0022 **	−0.0005 **	0.00161	–0.0027	−0.002	−0.0009 ***
Joint significance of the three caregiving hours variables	***	***	***	***	*	***	***	***	>0.1	*	***

We report the marginal effect point estimate and the standard error. † The probit coefficient representing the association between labor force participation and caregiving hours after the threshold was calculated by summing the coefficients of CG and CG*CG^. Its *p*-value represents the joint significance of CG and CG*CG^. * *p*-value < 0.1, ** *p*-value < 0.05, *** *p*-value < 0.01.

**Table 5:** Subgroup analyses on Chinese men assuming a weekly caregiving threshold of 72 h.

Hukou Status			Educational Status		Household Income		Hukou Region (Economic Macro-Regions)			Hukou Region (City Tiers)	
Variables	Urban (*N* = 814)	Rural (*N* = 1454)	Below Middle School (*N* = 809)	Middle School or above (*N* = 1459)	Below the Median (*N* = 1013)	Equal or above the Median (*N* = 1255)	East (*N* = 823)	Central (*N* = 767)	West (*N* = 572)	Tier 2 or above (*N* = 627)	Below Tier 2 (*N* = 1571)
Caregiving hours before the threshold, per hour	–0.000263 (0.000938)	–0.00150 ** (0.000616)	–0.00293 *** (0.000823)	0.000144 (0.000683)	–0.00120 (0.000829)	–0.00103 (0.000650)	–0.00214 ** (0.000919)	–0.00194 ** (0.000797)	0.00115 (0.00115)	–0.00181 (0.00110)	–0.00104 * (0.000612)
Discontinuity at the threshold of caregiving	0.816 * (0.439)	0.170 (0.216)	0.297 (0.317)	0.392 (0.273)	0.0913 (0.276)	0.969 ** (0.421)	0.196 (0.353)	0.295 (0.334)	0.568 (0.430)	0.970* (0.584)	0.248 (0.217)
Interaction between threshold and caregiving	−0.00728 ** (0.00361)	−0.000591 (0.00197)	−0.00146 (0.00287)	−0.00329 (0.00236)	−7.66e–05 (0.00247)	−0.00727 ** (0.00336)	0.000409 (0.00328)	−0.00136 (0.00283)	−0.00680 * (0.00387)	−0.00743 (0.00507)	−0.00154 (0.00193)
Caregiving hours after the threshold, per hour †	−0.00754	−0.00209	−0.00439	−0.00315	−0.00128	−0.0083 *	−0.00173	−0.0033	−0.00565	−0.00924	−0.00258
Joint significance of the three caregiving hours variables	>0.1	**	***	>0.1	>0.1	**	>0.1	**	>0.1	*	>0.1

We report the marginal effect point estimate and the standard error. † The probit coefficient representing the association between labor force participation and caregiving hours after the threshold was calculated by summing the coefficients of CG and CG*CG^. Its *p*-value represents the joint significance of CG and CG*CG^. * *p*-value < 0.1, ** *p*-value < 0.05, *** *p*-value < 0.01.

**Table 6:** Analyses that assess the relationship between LFP with hours of childcare and with hours of eldercare.

	Grandchild Care		Eldercare	
Variables	Women (Threshold = 4 h)	Men (Threshold = 72 h)	Women (Threshold = 7 h)	Men (Threshold = 70 h)
Caregiving hours before the threshold, per hour	−0.101 (0.0632)	−0.00199 *** (0.000627)	0.365 ** (0.149)	0.0218 * (0.0120)
Discontinuity at the threshold of caregiving	−0.00416 (0.0472)	0.423 * (0.223)	−0.127 (0.455)	−7.231 (12.39)
Interaction between threshold and caregiving	0.0985 (0.0633)	−0.00266 (0.00200)	−0.366 ** (0.147)	0.0434 (0.136)
Caregiving hours after the threshold, per hour †	−0.0025	−0.00465 *	−0.001 **	0.0652 **
Joint significance of the three caregiving hours variables	***	***	*	**

We report the marginal effect point estimate and the standard error. The one-step probit analysis results are reported for the grandchild care analysis while the instrumental variables analysis results are reported for the eldercare analysis. † The probit coefficient representing the association between labor force participation and caregiving hours after the threshold was calculated by summing the coefficients of CG and CG*CG^. Its *p*-value represents the joint significance of CG and CG*CG^. * *p*-value < 0.1, ** *p*-value < 0.05, *** *p*-value < 0.01.

## Data Availability

Publicly available datasets were analyzed in this study. These data can be found here: http://charls.pku.edu.cn/pages/data/2011-charls-wave1/en.html.

## Appendix A

**Table A1:** Results of a two-stage least-square and a limited-information maximum likelihood procedure using the proposed instruments.

	Two-Stage Least-Square		Limited-Information Maximum Likelihood	
Variables	Women (Threshold = 8-h)	Men (Threshold = 72 h)	Women (Threshold = 8-h)	Men (Threshold = 72 h)
Caregiving hours before the threshold, per hour, CG	0.117 (0.160)	0.00727 (0.00792)	−4.369 (20.05)	0.00810 (0.00843)
Discontinuity at the threshold of caregiving, CG^	0.454 (0.483)	−1.973 (3.812)	11.71 (48.60)	−2.166 (4.084)
Interaction between threshold and caregiving, CG*CG^	−0.128 (0.156)	−0.00258 (0.0350)	4.141 (19.12)	−0.00264 (0.0375)
Caregiving hours after the threshold, per hour †	−0.011	0.00469	−0.228	0.00546
Age, per one-year increase	−0.0291 *** (0.00669)	−0.0169 *** (0.00329)	0.0488 (0.359)	−0.0168 *** (0.00340)
Middle school	0.0141 (0.0481)	0.0205 (0.0272)	−1.092 (4.868)	0.0213 (0.0282)
High school	0.0326 (0.0463)	0.0583 * (0.0303)	−0.214 (1.201)	0.0581 * (0.0313)
College and above	0.237 ** (0.106)	0.0271 (0.0512)	1.714 (6.854)	0.0258 (0.0529)
Having work-limiting health conditions	0.395 *** (0.0605)	0.281 *** (0.0397)	0.758 (1.957)	0.284 *** (0.0415)
Urban residence	0.0196 (0.0433)	0.0908 *** (0.0255)	−0.939 (4.246)	0.0916 *** (0.0264)
Household size	−0.00193 (0.0174)	0.0188 * (0.0105)	0.0419 (0.342)	0.0192 * (0.0109)
Monthly spousal income (log-transformed)	0.0150 *** (0.00393)	0.0120 *** (0.00399)	−0.0167 (0.145)	0.0120 *** (0.00413)
Joint significance of the three caregiving hours variables	*	>0.1	>0.1	>0.1
**Tests establishing the validity of instruments**				
Anderson canon. corr. LM statistic (Under-identification test)	5.664 (*p*-value > 0.1)	8.418 (*p*-value > 0.1)	5.664 (*p*-value > 0.1)	8.418 (*p*-value > 0.1)
Cragg–Donald Wald F-statistic (Weak IV test)	0.803	1.198	0.803	1.198
Sargan statistics (Overidentification test)	16.37 (*p*-value < 0.01)	0.665 (*p*-value > 0.1)	5.611 (*p*-value > 0.1)	0.645 (*p*-value > 0.1)
Endogeneity test	5.822 (*p*-value > 0.1)	5.257 (*p*-value > 0.1)	5.822 (*p*-value > 0.1)	5.257 (*p*-value > 0.1)

We report the marginal effect point estimate and the standard error. We omitted the variable representing individual’s marital status due to multicollinearity. † The probit coefficient representing the association between labor force participation and caregiving hours after the threshold was calculated by summing the coefficients of CG and CG*CG^. Its *p*-value represents the joint significance of CG and CG*CG^. IV, instrumental variables. * *p*-value < 0.1, ** *p*-value < 0.05, *** *p*-value < 0.01.

## Appendix B

**Table A2:** Comparing individual characteristics stratified by gender and by the threshold of weekly unpaid caregiving hours.

	Women			Men		
Variables	CG < 8 (*N* = 970)	CG ≥ 8 (*N* = 480)	*p-*Value	CG < 72 (*N* = 2181)	CG ≥ 72 (*N* = 87)	*p*-Value
Labor force participation	0.53 (0.50)	0.35 (0.48)	***	0.76 (0.43)	0.67 (0.47)	***
Unpaid caregiving, hr/wk	0.44 (1.34)	52.49 (39.08)	***	6.58 (15.5)	112.75 (25.9)	***
Age, yr	49.4 (3.2)	50.5 (3.1)	***	52.2 (4.7)	54.2 (4.3)	***
Currently married	0.96 (0.20)	0.92 (0.27)	***	0.96 (0.20)	0.97 (0.18)	>0.1
Illiterate or primary school	0.45 (0.50)	0.52 (0.50)	>0.1	0.36 (0.48)	0.39 (0.49)	>0.1
Middle school	0.27 (0.44)	0.26 (0.44)	>0.1	0.35 (0.47)	0.37 (0.49)	>0.1
High school	0.23 (0.42)	0.20 (0.40)	>0.1	0.24 (0.43)	0.21 (0.41)	>0.1
College and above	0.05 (0.21)	0.02 (0.13)	**	0.06 (0.24)	0.03 (0.18)	*
Having work-limiting health conditions	0.12 (0.32)	0.07 (0.26)	**	0.13 (0.34)	0.21 (0.41)	***
Urban residence	0.36 (0.48)	0.37 (0.48)	>0.1	0.43 (0.50)	0.47 (0.50)	>0.1
Household size	3.4 (1.3)	4.1 (1.6)	***	3.7 (1.6)	4.6 (2.0)	***
Monthly income of spouse, RMB	1572.5 (2849.3)	1250.4 (2067.0)	*	452.9 (1355.0)	168.8 (547.5)	>0.1

We report the mean and the standard deviation. CG, caregiving; hr, hours; wk, weeks. * *p*-value < 0.1, ** *p*-value < 0.05, *** *p*-value < 0.01.

## Appendix C. Analyses Stratified by Types of Caregiving (Grandchild Care vs. Eldercare)

**Table A3:** Results of an association between labor force participation and weekly hours of grandchild care among women and men.

	One-Step Probit Model		Instrumental Variables Method in a LIML Procedure	
Variables	Women (threshold = 4 h)	Men (threshold = 72 h)	Women (threshold = 4 h)	Men (threshold = 72 h)
Caregiving hours before the threshold, per hour, CG	−0.101 (0.0632)	−0.00199 *** (0.000627)	−3.385 (5.396)	−0.0207 (0.0190)
Discontinuity at the caregiving threshold, CG^	−0.00416 (0.0472)	0.423 * (0.223)	7.969 (14.05)	17.39 * (10.02)
Interaction between threshold and caregiving, CG*CG^	0.0985 (0.0633)	−0.00266 (0.00200)	3.243 (5.176)	−0.113* (0.0681)
Caregiving hours after the threshold, per hour †	−0.0025	−0.00465 *	−0.142	−0.134
Age, per one-year increase	−0.0272 *** (0.00391)	−0.0178 *** (0.00201)	−0.0460 (0.0488)	−0.0181 *** (0.00590)
Married	−0.157 *** (0.0592)	0.0794 * (0.0416)	−0.554 (0.830)	0.0834 (0.103)
Middle school	0.0354 (0.0323)	0.0121 (0.0225)	−0.153 (0.377)	−0.0364 (0.0631)
High school	0.0892 ** (0.0368)	0.0460 * (0.0251)	−0.254 (0.650)	0.0292 (0.0675)
College and above	0.330 *** (0.0717)	0.0394 (0.0448)	−0.183 (1.029)	0.0815 (0.107)
Having work-limiting health conditions	0.504 *** (0.0544)	0.493 *** (0.0637)	0.129 (0.685)	0.202 ** (0.0843)
Urban residence	0.0178 (0.0285)	0.0791 *** (0.0193)	−0.227 (0.447)	0.0214 (0.0575)
Household size	−0.00377 (0.00896)	0.0119 ** (0.00588)	−0.0546 (0.128)	0.0116 (0.0190)
Monthly spousal income (log-transformed)	0.0179 *** (0.00333)	0.0142 *** (0.00340)	0.000518 (0.0380)	0.00939 (0.00790)
Joint significance test of the three caregiving variables	***	***	>0.1	>0.1
**Tests establishing the validity of the instruments**				
Anderson canon. corr. LM statistic (Under-identification test)	--	--	7.679 (*p*-value > 0.1)	9.743 (*p*-value > 0.1)
Cragg–Donald Wald F-statistic (Weak IV test)	--	--	0.690	0.881
Sargan statistic (Overidentification test)	--	--	7.334 (*p*-value > 0.1)	7.129 (*p*-value > 0.1)
Endogeneity test	--	--	5.736 (*p*-value > 0.1)	6.095 (*p*-value > 0.1)

We report the marginal effect point estimate and the standard error. † The probit coefficient representing the association between labor force participation and caregiving hours after the threshold was calculated by summing the coefficients of CG and CG*CG^. Its *p*-value represents the joint significance of CG and CG*CG^. LIML, limited-information maximum likelihood; IV, instrumental variables. * *p*-value < 0.1, ** *p*-value < 0.05, *** *p*-value < 0.01.

**Table A4:** Results of an association between labor force participation and weekly hours of eldercare among women and men.

	One-Step Probit Model		Instrumental Variables Method in a LIML Procedure	
Variables	Women (Threshold = 7 h)	Men (Threshold = 70 h)	Women (Threshold = 7 h)	Men (Threshold = 70 h)
Caregiving hours before the threshold, per hour, CG	0.0389 *** (0.0132)	0.00225 * (0.00115)	0.365 ** (0.149)	0.0218 * (0.0120)
Discontinuity at the caregiving threshold, CG^	−0.0681 (0.0479)	−0.280 (0.222)	−0.127 (0.455)	−7.231 (12.39)
Interaction between threshold and caregiving, CG*CG^	−0.0383 *** (0.0133)	0.000514 (0.00255)	−0.366 ** (0.147)	0.0434 (0.136)
Caregiving hours after the threshold, per hour †	0.0006 ***	0.00276	−0.001 **	−0.0216 **
Age, per one-year increase	−0.0352 *** (0.00391)	−0.0175 *** (0.00188)	−0.0446 *** (0.00715)	−0.0201 *** (0.00484)
Married	−0.0923 (0.0631)	0.0979** (0.0392)	−0.108 (0.101)	0.159 ** (0.0625)
Middle school	0.0168 (0.0340)	−0.00206 (0.0220)	0.0308 (0.0453)	−0.0147 (0.0305)
High school	0.0890 ** (0.0364)	0.0369 (0.0237)	0.0396 (0.0510)	0.0104 (0.0355)
College and above	0.316 *** (0.0575)	0.0257 (0.0399)	0.196 * (0.103)	−0.0116 (0.0562)
Having work-limiting health conditions	0.541 *** (0.0539)	0.407 *** (0.0557)	0.378 *** (0.0617)	0.213 *** (0.0373)
Urban residence	0.0407 (0.0305)	0.0703 *** (0.0187)	0.0728* (0.0440)	0.0738 *** (0.0257)
Household size	−0.00766 (0.0101)	0.00882 (0.00581)	−0.00195 (0.0145)	0.00706 (0.00913)
Monthly spousal income (log-transformed)	0.0168 *** (0.00345)	0.0121 *** (0.00306)	0.0149 *** (0.00519)	0.00802 (0.00512)
Joint significance test of the three caregiving variables	***	>0.1	*	**
**Tests establishing the validity of the instruments**				
Anderson canon. corr. LM statistic (Under-identification test)	--	--	12.36 (*p*-value < 0.05)	2.170 (*p*-value > 0.1)
Cragg–Donald Wald F statistic (Weak IV test)	--	--	1.758	0.308
Sargan statistic (Overidentification test)	--	--	2.267 (*p*-value > 0.1)	1.093 (*p*-value > 0.1)
Endogeneity test	--	--	8.584 (*p*-value < 0.05)	11.61 (*p*-value < 0.01)

We report the marginal effect point estimate and the standard error. † The probit coefficient representing the association between labor force participation and caregiving hours after the threshold was calculated by summing the coefficients of CG and CG*CG^. Its *p*-value represents the joint significance of CG and CG*CG^. LIML, limited-information maximum likelihood; IV, instrumental variables. * *p*-value < 0.1, ** *p*-value < 0.05, *** *p*-value < 0.01.
